# Beyond the Medication Pass: Attitudes, Ethics, Agency, and Antipsychotic Medications in Assisted Living/Residential Care

**DOI:** 10.1093/geroni/igac052

**Published:** 2022-08-17

**Authors:** Sarah Dys, Paula Carder

**Affiliations:** School of Public Health, Oregon Health and Science University–Portland State University, Portland, Oregon, USA; Institute on Aging, College of Urban and Public Affairs, Portland State University, Portland, Oregon, USA; School of Public Health, Oregon Health and Science University–Portland State University, Portland, Oregon, USA; Institute on Aging, College of Urban and Public Affairs, Portland State University, Portland, Oregon, USA

**Keywords:** Behaviors, Home- and community-based care, Situational analysis, Medication management, Morality

## Abstract

**Background and Objectives:**

As-needed (PRN) antipsychotic medication use (APU) among assisted living/residential care (AL/RC) residents is a controversial health policy issue. AL/RC care staff, families, clinicians, researchers, and policymakers disagree about PRN APU to manage behavioral expressions associated with residents’ dementia or cognitive impairment.

**Research Design and Methods:**

Semistructured interviews among eleven direct care staff (*n* = 3), licensed nurses (*n* = 2), administrators/executive directors (*n* = 4), and consultant pharmacists (*n* = 2) currently working in Oregon AL/RC. Using situational analysis, we identify, describe, and visualize positions and ideologies by job role to theorize PRN APU decision-making.

**Results:**

Three broad processes underlie APU to manage residents’ behavioral expressions: justifying PRN APU, moralizing APU, and balancing local practices (eg, managing behavioral expressions, respecting individuals) with nonlocal practices (eg, professional authority). People involved in the situation of APU in AL/RC describe positive and negative justifications, and personal moral positions that frame PRN antipsychotics or nonpharmaceutical interventions as “right” or “wrong,” driving various approaches to behavior management. Participants described a converse orientation between perceived level of agency within and proximity to the situation of APU. Those most closely involved, or local, to the situation of passing medications (eg, direct care staff and nurses) expressed less agency compared with nonlocal physicians and policymakers, who are not involved in the day-to-day practices within AL/RC.

**Discussion and Implications:**

This study raises practice and policy implications regarding APU in AL/RC settings. Care staff roles, ethical considerations, and perceived agency inform decision-making on whether to use antipsychotic medications. Participants described costs and benefits associated with both PRN APU and nonpharmaceutical interventions when responding to AL/RC residents’ behavioral expressions. Participants’ experiences emphasize the interactions across multiple levels of care. Balancing regulatory goals with resident-centered practices underscores the need for a system-level perspective, extending beyond direct care staff passing antipsychotic medications to residents.

**Translational Significance:** This study highlights the complex, multilevel nature of antipsychotic medication use in AL/RC contexts, where values, regulatory oversight, and resident centeredness might not align cohesively in practice. AL/RC staff are forced to simultaneously balance and prioritize regulatory goals, organizational constraints, and complex care provision resulting in a multilayered, difficult, and unique situation. This study addresses how system-level conceptualization of antipsychotic medication use in AL/RC has implications to improve person-centered care in AL/RC. Lessons learned can guide a reimagining of antipsychotic medication use in older adults living with dementia in home- and community-based services settings and improve care practices.

## Background and Objectives

Assisted living and residential care (AL/RC) is a significant segment of the long-term services and supports sector in the United States (1,2). Of the estimated 918 700 AL/RC residents in the United States, 49% have an Alzheimer’s disease or related dementia (ADRD) diagnosis (3). Over 70% of AL/RC residents living with ADRD or cognitive impairment experience behavioral expressions (4). Behavioral expressions can include aggression, agitation, anxiety, delusions, hallucinations, and sleeplessness (5,6). Chronic or severe behavioral expressions have implications for residents’ quality of life, family and caregiver burden, and care transitions (7,8).

Existing guidelines and practices encourage psychosocial or environmental interventions as the first line of treatment in managing behavioral expressions as a person-centered, safe, alternative to medication (9–11). When nonpharmaceutical interventions fail or a resident is in considerable, persistent distress, pharmaceutical management of behaviors using psychotropic medication may be appropriate (11). Psychotropic medications interact with the central nervous system and include the following drug classes: antipsychotics, antidepressants, anxiolytics, hypnotics, and mood stabilizers (12).

Though every psychotropic medication class is associated with significant risks in older adult populations (13–16), antipsychotic medications have received explicit empirical and regulatory attention. In the early 2000s, studies demonstrated that off-label use of antipsychotic medications in older adults with dementia was associated with a higher risk of early mortality (17,18), leading the U.S. Food and Drug Administration to issue a “black box” warning on antipsychotic medication use (APU) in older adults (19). Given the prevalence of older adults living with dementia in long-term residential care settings, the Centers for Medicaid and Medicare Services developed quality improvement efforts related to the use of these medications, forming the National Partnership for Quality Dementia Care (20), which has been associated with reductions in APU in nursing home populations over time (21,22). There is still lack of evidence regarding how antipsychotic medications are used, staff training and implementation of nonpharmaceutical interventions, uptake of other psychotropic medications beyond antipsychotics, or potential discrimination against older adults whose behaviors are deemed “challenging” within the AL/RC context.

Among many reasons antipsychotic medications are used among older adults living with ADRD, behavioral expressions labeled as agitation and aggression are common indications (6,23). Clinicians, direct care staff, and family members reportedly view APU as positive, safe, and effective (23,24), though much of the existing literature only focuses on nursing home residents and staff. One qualitative study detailed how primary care providers view pharmaceutical responses to behavioral expressions associated with ADRD as less risky than empirical evidence suggests and that although policies do successfully decrease APU, they unintentionally promote other, riskier medications (24). The same study team found direct care staff and family caregivers in nursing homes described significant systemic and interpersonal barriers to performing nonpharmaceutical interventions compared with the effectiveness of medications (25).

Key factors influencing decision-making around APU in nursing homes include organizational capacity, individual professional capability, communication and collaboration, attitudes, and regulations/guidelines (26). In comparison to routine/scheduled use of these medications, *pro re nata* (PRN; as needed) medication orders to respond to behavioral expressions presents an additional layer of complexity and decision-making, especially for direct care staff who are not permitted to formally assess residents’ needs (27–29).

Direct care staff (eg, caregivers, certified nursing assistants, and medication aides) build, maintain, and negotiate relationships with their residents, learning from and basing care decisions on residents’ cognitive, physical, and emotional cues on a daily basis (27,30–32). For those staff that assists with medications administered “as-needed,” knowing individual residents’ behaviors and nuances is critical to decision-making (6,27,30,31,33). Direct care staff may employ several strategies when identifying and responding to behaviors, such as redirection, isolation, seeking assistance, or communicating with external care providers (6). AL/RC care models that prioritize autonomy, choice, dignity, independence, individuality, and privacy introduce another component to the equation of medication management and APU (31–33). Balancing these espoused values (eg, autonomy) with the mandate to protect residents (eg, safety) can result in multiple sources and levels of conflict regarding an “appropriate response” to residents’ behavioral expressions and medication needs (32).

APU in AL/RC settings is an understudied and controversial community health and policy issue, and less is known about PRN use of these medications. To address this gap, we examine decision-making related to administering PRN antipsychotic medications among several types of staff who have first-hand experience in AL/RC settings: direct care workers, nurses, administrators, and consultant pharmacists.

### Research Design and Methods

The overall study design was situational analysis (34), which extends grounded theory by identifying, conceptualizing, analyzing, and visualizing *situations* that construct processes occurring within a social world (34). Existing theories about PRN medication use in LTSS focus on quality and compliance, using institutional and organizational theories (29). Our approach builds on prior examinations of AL/RC as a social world based on interpersonal interactions (31,33), including a situational analysis of medication management (33), to develop a working theory grounded in daily practice. We used the constant comparative method during data collection and analyses by conducting interviews, transcribing, reading, and preparing inductive codes that iteratively informed subsequent data collection and analyses (35,36). Our reporting follows guidelines outlined by the COnsolidated criteria for REporting Qualitative research (COREQ) (37).

### Stakeholder Advisory Board

Due to their contributions to and familiarity with Oregon’s sociohistorical AL/RC policy and practice context, we invited representatives from key organizations including the Oregon Department of Human Services/Aging and Persons with Disabilities (DHS/APD), Oregon Health Authority, Quality Metrics Council, Oregon Partnership for Quality Dementia Care, and AL/RC providers to form a stakeholder advisory board for this research. Eight committee members consisted of community-based care providers, geriatric-trained clinicians with long-term care experience, state agency representatives, and researchers regularly provided feedback on interview guide development, sampling, recruitment, and initial findings.

### Sampling and Recruitment

Using a publicly available DHS/APD list of all licensed AL/RC communities in Oregon as of January 2021 (*n* = 535) (38), we began with a maximum variation approach to recruit settings based on geography (rural/urban), profit status (for-profit/nonprofit), Medicaid acceptance (yes/no), and license type (AL/RC/MC). To reduce the number of settings to a manageable size, we started with a random number generator to identify an initial 25 communities sorting them by geography, profit status, Medicaid acceptance, and license type to begin recruitment. Between April and June 2021, the first author emailed a flyer explaining the study’s purpose and asked administrators to both participate in interviews and disseminate the flyer to their employees to self-select into the study. This flyer contained an internet hyperlink and QR code linked to a digital consent form and interview scheduling tool, and a brief survey of participants’ age, gender, race/ethnicity, and job title. We also implemented stakeholder advisory board members’ input by sending our flyer to executives of a healthcare company that provides consultant pharmacy services to AL/RC settings in Oregon for distribution.

The coronavirus pandemic posed a significant barrier to recruitment (see Limitations). In consultation with the stakeholder advisory board, we pivoted to a convenience sampling approach. We sent recruitment flyers and emails to a total of 130 AL/RC community administrators and one large healthcare company. Of these, 7.7% indicated they needed permission from corporate management to participate and did not respond to phone follow-up, 24.6% declined to participate in the study on behalf of their communities, 29.2% responded they would post the recruitment flyer in their communities, 38.5% did not respond to email solicitation or phone follow-up. Nineteen individuals filled out the online demographic survey, 16 consented and scheduled an interview, and 11 completed an interview between May and August 2021. These 11 participants included four administrators/executive directors, 3 unlicensed direct care workers, 2 consultant pharmacists, 1 licensed professional nurse, and 1 registered nurse/resident care coordinator. Our decision to stop data collection after eleven interviews involved reflexive open coding and discussion as interviews were being collected, data interpretation through guided mapping exercises dictated by situational analysis, and pragmatism required to conduct this work during the coronavirus pandemic (39,40). Participants received a $20 Amazon gift card. This study was approved by Portland State’s Institutional Review Board (protocol #: 206858-18).

### Data Collection

Both authors have publication records and a combined 25 years of experience conducting in-depth interviews and focus groups with AL/RC residents, staff, and stakeholders within Oregon and across the United States. The first author, a research associate with a doctorate in public health specializing in gerontology, was trained by the second author, a professor in public health and nationally recognized expert in AL/RC policy and qualitative research methods, including situational analysis.

The first author conducted all interviews and met with the second author biweekly during data collection to debrief findings, begin constructing analytic codes, and develop evolving iterations of maps used to facilitate situational analysis. Interviews ranged from 28 minutes to 46 minutes (average 35 minutes), took place over the phone, were recorded, and transcribed over Zoom. Participants provided additional, verbal consent to recording and then proceeded with the interview. We used a semistructured interview approach (Supplementary Material 1), beginning each interview with an introduction of the study scope and three questions:

1) Please describe your job and responsibilities.
2) Thinking about residents who express behaviors, tell me about a time when one of those residents was helped through successful management by you and other staff?
3) How about a resident whose behavioral expressions were so severe that you or your staff were not able to respond, can you tell me that story?

We designed these questions based on a recent study examining staff reports of residents’ behavioral expressions in AL/RC settings (6). How a participant described their roles and responsibilities guided follow-up from the available question bank. For example, a self-qualified medication aide described medication passing as one of their roles, leading to questions about their experiences administering PRN antipsychotic medications. However, if direct care staff indicated they did not have a role in medication administration, administration questions were not asked. The experience participants chose to share also facilitated additional probes and follow-up questions during the interview.

### Data Analysis

The situation of interest, and analytic unit, is descriptions of how and why antipsychotic medications are used within AL/RC settings. These descriptions include human (eg, staff, residents, prescribers) and nonhuman (eg, antipsychotic medication, training, medication records) actors that interact together to negotiate social processes around medication administration (33). We begin with traditional grounded theory methods for analyzing qualitative data, applying constant comparison to iteratively develop and examine codes, writing analytic memos on emerging topics and patterns, and theoretical saturation through mapping (34–36,40). By open coding the first transcribed interview, we identified initial descriptions of PRN APU, and general code categories including roles (eg, people, positions, jobs identified as involved), attitudes (eg, emotions, positions, perspectives related to APU and/or behavioral expressions), processes (eg, descriptions of what leads to PRN antipsychotic medication administration), and proximity (eg, in relation to AL/RC residents and receiving antipsychotic medication, where are roles, attitudes, and process situated?).

**Figure 1.**
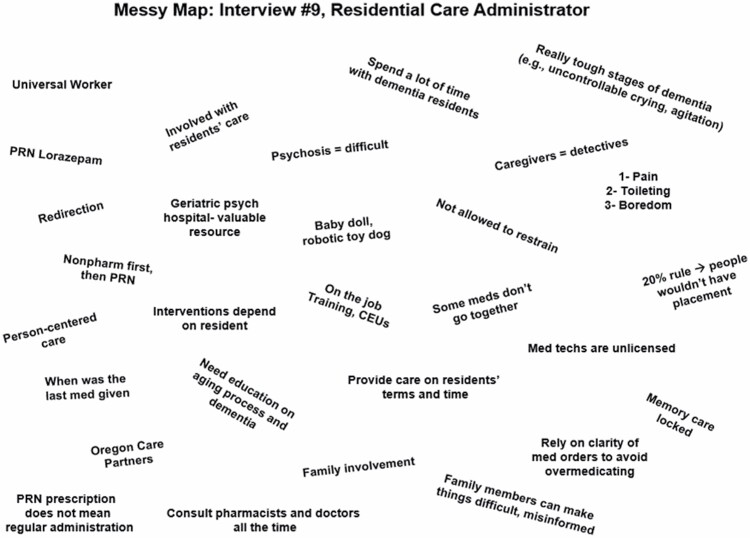
An example of a messy map constructed of interviewee #9.

Situational analysis seeks to identify many possible interacting elements and characteristics related to a phenomenon of interest through a systematic series of visualization exercises, or mapping (34). This process supported axial and selective coding of local and nonlocal elements (41) that inform decision-making related to PRN APU from the perspective of various actors within AL/RC settings. We used paper, pencil, and templates available through SAGE Publishing to generate maps (42). Using analytic memos to reflect on the emergence of topics within interviews and potential relationships among these topics (eg, axial coding), we developed “messy maps” (40) for each interview (Figure 1). We used messy maps to capture the breadth of topics, elements, and perspectives raised during interviews. Through an iterative process of analytic memoing and identification of preliminary themes, we continued to integrate analytic and theoretical relationships, generating numerous maps. We used meetings and map development to determine a stopping point in recruitment, as interviews did not yield novel theoretical development related to answering our research question. Next, we developed an ordered situational map to organize elements identified, during messy mapping, including those that might be unstated, or “silent” (Supplementary Material 2) (34).

The purpose was to identify elements that illustrate and contextualize the situation of PRN APU within AL/RC settings based not only on interviews, but also primary human (eg, residents, staff) and nonhuman (eg, PRN medications, medication records, order parameters, behaviors) elements, collective groups or organizations, discourses constructing human and nonhuman actors, political, sociocultural, temporal, historical, and other pertinent situation characteristics (34,40).

Participants described numerous human and nonhuman elements, identified positions and relationships among these entities with external political and social beliefs, and expressed varying positions and decision-making related to PRN APU. We combined maps that categorized these elements with line-by-line interview coding (33,34) and wrote thematic statements that form the foundation of theorizing (35,43). We plotted these descriptions along axes, using codes and quotes to visualize positions and relationships among human and nonhuman elements identified by participants. In sum, this process of traditional coding and memoing, combined with messy and positional mapping, anchored theory development (34).

## Results

### AL/RC Settings and Interview Participants

Tables 1 and 2 describe participants’ demographic characteristics and the settings where they worked: five separate AL/RC communities and one company providing consultant pharmacy services. Participants’ roles included administrators/executive directors (*n* = 4), unlicensed direct care workers (*n* = 3), consultant pharmacists (*n* = 2), a licensed professional nurse (*n* = 1), and a registered nurse/resident care coordinator (*n* = 1). Two unlicensed direct care workers self-qualified as medication aides or technicians. In Oregon, medication administration is defined as a role for direct care workers, a certification is not required (44).

**Table 1.** Descriptive Characteristics of Interview Participants (*n* = 11)

Individual Characteristics	Mean (Range)	*n*
Age, y	44 (27–64)	
Race/ethnicity		
Non-Hispanic White		7
Asian American or Pacific Islander		2
Multiracial		1
Not disclosed		1
Gender		
Woman		7
Man		3
Gender nonconforming		1

**Table 2.** Descriptive Characteristics of Settings (*n* = 6)

Setting Description	Accepts Medicaid	Number of Beds	Participant Role
A: Stand-alone MC, rural, not-for-profit	Yes	32	1 Administrator
B: RC, urban, for profit	Yes	70	1 Administrator, 1 licensed professional nurse
C: RC/MC, urban, for profit	Yes	55	1 Executive director, 2 unlicensed direct care workers
D: RC, urban, for profit	Yes	15	1 Administrator, 1 unlicensed direct care worker
E: AL/MC, rural, for profit	Yes	50	1 Registered nurse
F: Provides consultant pharmacy services to long-term care settings across the state	N/A	N/A	2 Consultant pharmacists

*Notes:* AL = Assisted living, MC = Memory care, RC = Residential care.

We theorize that decisions to administer antipsychotic medications involve ongoing negotiations framed by the individual actors’ authority and proximity to the situation. These negotiations manifest through three mechanisms identified through situational and positional mapping. These include justifying PRN APU, moralizing responses to residents’ behavioral expressions, and balancing local and nonlocal practices and perceived authority.

### Justifying As-Needed Antipsychotic Medication Use

**Figure 2.**
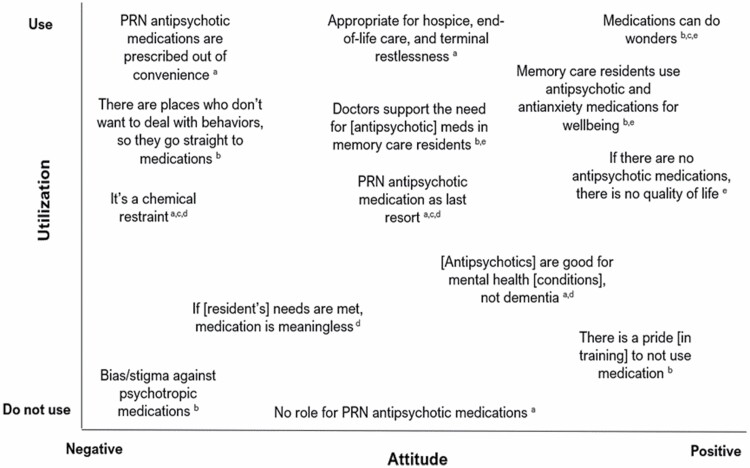
Positional map of expressed ideologies on PRN antipsychotic medication use in assisted living/residential care residents with dementia. Superscripts identify the roles of actors who expressed the positions: ^a^Consultant pharmacist; ^b^administrator/executive director; ^c^unlicensed direct care worker; ^d^licensed professional nurse; ^e^registered nurse/resident care coordinator.

Interview participants described PRN APU, types of related training, and how a hypothetical utilization threshold (ie, 20% resident population; Supplementary Material 1) at the policy level would affect their work. When answering, participants conveyed different attitudes and beliefs regarding PRN APU in people with dementia living in AL/RC settings. One direct care worker (Participant 11) stated that “*medication is an emotional, heavy topic*” to discuss. Figure 2 depicts the range of attitudes expressed by interviews and their relationship with whether to use or not use antipsychotic medication; participant roles are denoted in figure notes.

When describing examples of behavioral management, participants tended to describe whether it was appropriate to use or not use PRN antipsychotic medications. Participants underscored their comments and stories with positive, negative, and/or neutral attitudes regarding APU.

Nearly every participant iterated in some capacity that medications are effective when residents exhibit behavioral expressions, pain, and/or other discomfort. Despite all efforts to address residents’ needs and respond to behavioral expressions, residents may not respond to nonpharmaceutical interventions. Several direct care staff shared protocols and processes that justify behavioral response in the communities where they work. After a number of unsuccessful nonpharmaceutical interventions, they rely on the resident’s care plan and facility policy to use medications as one self-identified medication aide (Participant 7) described,

[We use] at least three interventions usually laid out, like, planned interventions things that might work: snacks, toileting, repositioning. And then talking to the family about their [the resident’s] past and getting ideas to redirect them. […] But if at least three attempts don’t work usually for the course of at least half an hour that’s when we would consider giving them something stronger, some medication.

Though interview participants with direct care roles were asked to describe situations where they administered PRN antipsychotic medications to residents, few shared stories about antipsychotic medications, specifically. When describing medications’ effectiveness, interviewees often discussed PRN medications overall, including other types of medications, “*Yeah PRN, so you’re talking about hydrocodone, oxycodone, lorazepam, morphine, you know?*” (Participant 9). When asked to describe a situation that necessitated PRN antipsychotic administration, nearly every participant (outside of consultant pharmacists) described an antianxiety medication administration (eg, lorazepam). This suggests a conceptualization of medications based on what they are commonly used to treat.

Direct care staff, registered nurses, and administrators each justified using medications that promote residents’ comfort and quality of life. Two licensed nurses (Participants 5 and 10) explained that AL/RC residents with PRN medication orders are able to self-direct and ask for those medications. However, for residents in memory care settings,

[…] a lot of the residents use antipsychotics and antianxiety [medications] because it’s for their wellbeing and they’re up, they’re functional, they’re going to activities, they have a good appetite, they’re socializing, they’re interested in things, you know? They’re communicating to the best of their ability, they’re definitely having a good quality of life, but if they didn’t have those medicines they wouldn’t be having a good quality of life. (Registered Nurse, Participant 10)

Some direct care staff and those with pharmaceutical backgrounds stated as-needed antipsychotic (and other psychotropic) medications in residents living with dementia is never appropriate. Rather, using these types of medications functions as a restraint and indicates residents’ needs are not being met. A consultant pharmacist (Participant 3) who works with communities to conduct resident medication review said,

These drugs are all indicated to treat schizophrenia and various psychiatric disorders, but when we’re using them to treat dementia, they don’t do anything in dementia, they don’t slow its progression. We’re essentially using a chemical restraint. And sometimes that’s necessary somebody that has exacerbating behaviors and is explosive and hitting everybody, kicking scratching basically the neuropsychiatric symptoms of dementia, we don’t really have anything to treat those symptoms, so we default to antipsychotics. You use them basically as tranquilizers.

Overall, despite the job role, participants shared that PRN APU has appropriate and inappropriate uses for AL/RC residents. A combination of clinical decision-making, resident-driven care planning, and appropriate staffing were all cited as facilitators to making an appropriate decision whether to administer PRN antipsychotic medications.

### Moralizing Response to Residents’ Behavioral Expressions

**Figure 3.**
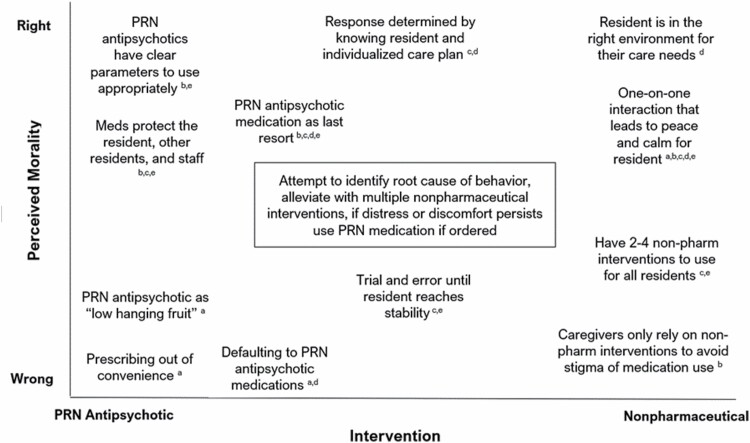
Positional maps of perceived morality associated with response to assisted living/residential care residents’ behavioral expressions. Text in the center box describes the prescribed rule via regulations and recommended practice. Superscripts identify the roles of actors who expressed the positions: ^a^Consultant pharmacist; ^b^administrator/executive director; ^c^unlicensed direct care worker; ^d^licensed professional nurse; ^e^registered nurse/resident care coordinator.

When asked how they responded to resident behaviors, either successfully or unsuccessfully, several individuals raised the premise of “responding right” or “responding wrong,” by which they meant morally right, as opposed to accurate. Participants acknowledged that responses should ideally center individual residents and their needs. However, respondents described an ethical toggle when deciding to use a nonpharmaceutical intervention versus a PRN antipsychotic medication. This toggle was framed in several ways (Figure 3). Generally, respondents oriented their perspectives to “medication as a last resort” through broadly applied nonpharmaceutical interventions or individually designed interventions. In one community, a self-qualified medication technician described typical nonpharmaceutical responses to residents’ behavioral expressions:

We have some residents with dementia and they yell, they scream, so sometimes they are hungry, we want to offer them snacks, you know? Maybe their briefs are wet, so we need to change [their briefs]. Or sometimes there’s some resident like to watch TV, so there’s loud music, you know, some people doesn’t want to hear it, so we need to change the, you know, place. Like, we need to bring [them to] their room, or we need to bring some things in and turn down the music, you know, like that. (Direct care staff, Participant 9)

Some participants detailed specific interventions developed for individual residents based on specific behaviors, resulting in successful redirection or behavior mitigation without PRN medications. An executive director (Participant 6) described a memory care resident who often attempted to leave the secured unit:

One woman, she ran dog shows, and so, she would travel to different parts of the country and put on dog shows, and there would be awards, and all of that, and so, she would believe oftentimes in the afternoons that she had to get out of here so that she could go catch a plane for a dog show somewhere. So her care plan listed specific things to say to her, because she was worried she was going to miss her plane, and so we would say, ‘Oh, my gosh I forgot to tell you it’s been rescheduled until tomorrow. You’re going to get your flight in the morning.’

Others shared that despite all best efforts, sometimes “medications just work” and one can “try everything” and residents will remain in distress. Participant 6 followed their example with a caveat and concern for the personal and professional preference towards nonpharmaceutical interventions, associating too many with negative consequences,

So lots of times we use, most times we use, nonpharmaceuticals. My personal concern is that, having been in long term care since ‘93, I see a lot of non-licensed staff who are taught to believe that there’s a concern that they’re overmedicating people or using those psychotropics for their convenience. And that bias that is built into them results in them trying too many non-pharmaceuticals, resulting in poor outcomes for our folks.

Interview participants tended to describe multiple perspectives over the course of their interviews. Nearly every participant started by describing an overarching moral position regarding the “right or wrong” of medications versus nonpharmaceutical interventions. Concurrently, participants also justified decisions or situations that contradicted their primary view.

For example, a consultant pharmacist (Participant 2) stated that there is no role for PRN antipsychotic medications and followed with an exception to this rule,

Honestly there’s really no role for PRN antipsychotics, there are very rare circumstances. You know, there’s instances, maybe end-of-life care terminal restlessness. In general, for [PRN] antipsychotics, we call them low hanging fruit, we need to get rid of those. There’s very rare situations that we should use [them].

#### Weighing “good” versus “bad”

Several participants indicated they had either worked at or heard of settings that abuse PRN medications. One administrator of a memory care setting (Participant 1) shared the complexity around the issue of PRN APU among residents, saying,

It’s a really touchy subject because I am sure there are some places out there who overmedicate and they will do that because they don’t want to deal with the behaviors, which is a huge disservice to our people. So finding that balance is super important, you know you always hear the bad guys ruin it for the good guys. That’s true, because a lot of these policies out there, they don’t allow you to use them [medications] as they should be, in the correct manner, because people abuse it. But we are not all bad I promise you.

Perceptions of goodness also extend to trust in and beliefs about clinical providers and prescribers responsible for overseeing medication management. A direct care worker (Participant 11) raised moral concerns when speculating on the reasoning behind using medication to respond to behaviors,

Since the pill almost seems like a restraint, or I don’t want to say a punishment, but it’s like ‘we can’t handle you [resident] anymore, so we need you to take this pill, so we don’t have to, you know, deal with you’ is kind of what it feels like. But it can’t be that way right? Because there’s all these caring people, the administrator is really nice, the doctor is really nice, the nurse is really nice.

Participant 10 (registered nurse) described their perceptions of prescriber’s intentions and knowledge related to prescribing PRN antipsychotic medications to residents, suggesting this treatment modality is both well-intentioned and informed,

The people who are ordering the medications, I’m assuming they’re all good people and they wouldn’t just order things negligently, but I can say that they are aware enough of the pros and cons of antipsychotics.

### Balancing Local and Nonlocal Practices and Perceived Authority

**Figure 4.**
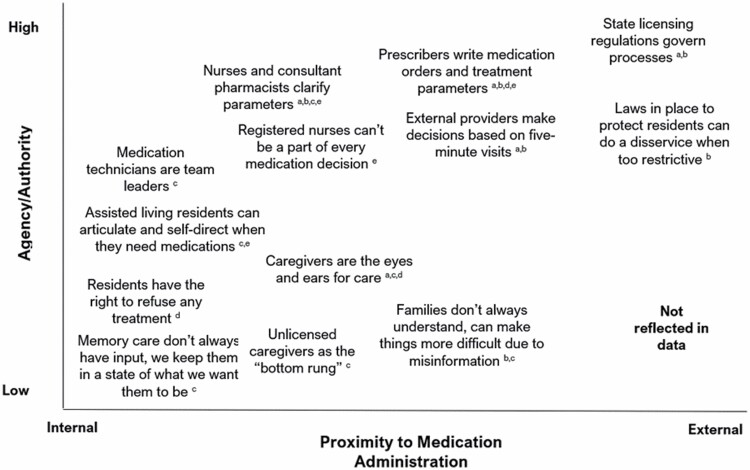
Positional map of perceived agency and proximity to PRN antipsychotic administration in assisted living/residential care settings. Superscripts identify the roles of actors who expressed the positions: ^a^Consultant pharmacist^; b^administrator/executive director; ^c^unlicensed direct care worker; ^d^licensed professional nurse; ^e^registered nurse/resident care coordinator.

Participants discussed the complexity surrounding PRN antipsychotic medication administration. By the time direct care staff administer a PRN antipsychotic medication, several actors with varying credentials, familiarity, and proximity to the situation and the resident have made several decisions. Participants oriented their descriptions of agency and authority to make decisions along a spectrum of local (ie, proximal) and nonlocal (ie, external) practices to the situation of PRN APU. The multilayered nature of PRN antipsychotic medication administrations suggests different power dynamics and ability to participate in the situation (Figure 4). As a consultant pharmacist (Participant 3) shared,

It [antipsychotic medication use] is really a prescribers’ issue. The facility doesn’t write the prescription. So the onus is on the facility to try to get gradual dose reduction or at least get a response and there’s a lot of physicians that just won’t respond. If you ever really wanted to change, you’d have to mandate some sort of physician training like they had to do with opioids or something like that. Yeah and probably get the state Board of Medical Examiner’s involved in mandating some sort of specialized CME [continuing medical education] or something.

Participants described different levels of agency depending on (a) residents’ level of cognitive impairment and ability to communicate and (b) staff’s roles within the settings. Participant 11 described their observations and resulting questions when giving residents with behaviors medications,

The pill almost seems like a restraint, or I don’t want to say a punishment, but it’s like where we can’t handle you anymore, so we need you to take this pill, so we don’t have to deal with you. [...] They give me a pill after [a resident] does these behaviors and then this is the outcome after [the resident] takes the pill, just there in [their] chair, you know? So like all the stuff that’s in my mind as a caregiver, and I don’t have the authority to say anything, or if I did have questions how would I address them or take them to my administrator or my nurse?

This participant expressed feeling disempowered to voice their concerns and ask questions by virtue of their position as an unlicensed direct care worker. Participants with medication-related roles tended to focus on the responsibility of medication administration,

At night I’m basically the acting supervisor. I make sure that all of the tasks are done and the caregivers are getting their jobs done as well as doing whatever scheduled medications there are and PRN medications, often helping with end of life comfort. (Direct care staff, Participant 7)

Participants also raised the importance of knowing and building relationships with residents to facilitate quality care and implement best practices. Staff turnover, low staffing ratios, and low perceived agency among staff within AL/RC settings compared to external providers were commonly expressed obstacles related to meeting residents’ needs and responding to behaviors.

Participants expressed both appreciation and frustration with existing and proposed regulations and policies. While appreciating the purpose of regulations to protect the safety and well-being of residents, participants feel those in the position to enact rules and requirements are disconnected from the practice of providing care to residents,

It is something that you wish, you know, the rule makers in the legislature and up at DHS [Department of Human Services] knew about the work that you do when they’re setting regulations. They know they can’t know every building in the whole state of Oregon, but it would be nice if they would ask for more information about the populations we deal with and what kind of difficulties we have. (Administrator, Participant 4)

State regulations set the minimum standards expected of AL/RC when providing care and services to residents. These rules and others involved in amending regulations are typically removed from the daily rhythm within AL/RC settings. Though not explicitly raised in interviews, other service providers external to medication administration within AL/RC settings (eg, social workers, psychiatric providers) might have input and agency related to APU.

## Discussion and Implications

We explored how those within the AL/RC setting with direct care connection and medication oversight of AL/RC residents, including direct care staff, medication aides, administrators, and consultant pharmacists, make decisions regarding PRN APU. Using situational analysis, we learned that attitudes (ie, positive, negative, and neutral), an underlying morality guiding interventions, and perceived agency within the context of medication administration influenced study participants’ ideologies around PRN APU. For those closest to the situation, such as direct care staff, the negotiation is informed by rules, training, and the needs of the person in front of them. For those further removed from the situation, such as consultant pharmacists, the negotiation is informed by professional standards, training, and an awareness of the challenges presented by some behavioral expressions. These findings highlight the complexity underlying APU in AL/RC settings and build upon narratives of care process negotiation within the AL/RC context (27,30–33).

The finding that positive, negative, and even neutral attitudes frame participants’ beliefs around antipsychotic medications used to respond to AL/RC residents’ behaviors is largely confirmatory. Like Kerns et al. found (24,25) participants with positive attitudes cited APU as largely effective and promote well-being, especially for residents living with dementia. Our participants’ views related to PRN APU situated along a positive/negative binary support Gill et al.’s study of scheduled antipsychotic medications (41). A pattern emerged between job roles and whether participants were more likely to express positive or negative attitudes toward APU. At one end of the spectrum, direct care staff and consultant pharmacists described how using PRN antipsychotic medication to respond to residents’ behaviors is effectively a chemical restraint for settings with staff that “don’t want to deal with them [residents].” On the opposite end of the spectrum, nurses, administrators, and self-identified medication aides were more likely to frame APU as promoting resident quality of life and well-being. Evaluations of the Halting Antipsychotic use in Long-Term care (HALT) study suggest that staff type plays a role in influencing the success or failure of antipsychotic medication deprescription (28,45). Combined with the context of one’s role within AL/RC, how participants conceptualize and perceive dementia and dementia care needs can influence care decisions (46,47).

Dementia care involves systems of thought and belief that guide decisions about what is “good” and “bad” (ie, morals) and what might be “right” or “wrong” to do (ie, ethics) (47,48). Furthermore, implications of and ethical issues with off-label APU to manage behavioral expressions have been discussed across the life course and globe (48–52). This conversation is further complicated by whether actors’ motivations align or conflict (32). An oversimplified example might manifest as clinicians pursuing therapeutic goals (eg, symptom management), families concern with safety goals, direct care staff aiming towards resident-centered goals, and administrative staff prioritizing compliance goals (50,53).

Participants’ experiences highlighted an underlying morality that partially drove whether to approach residents’ behavioral expressions with PRN antipsychotic medication or nonpharmaceutical interventions. Participants detailed examples of when it is “right” or “wrong” to use either PRN antipsychotic medications or nonpharmaceutical interventions. If nonpharmaceutical interventions are ineffective, and the resident remains in distress, PRN psychotropic medications may be considered (11,44,54). Major sources of moral distress for staff include understaffing, perceiving residents with dementia in pain, and not having enough time to provide adequate care (55).

Some participants raised concerns with providing care that might be “regulation-centered” as opposed to resident-centered, resulting in unintended consequences (56). Fear of regulatory noncompliance might lead staff to attempt too many behavioral interventions, prolonging a resident’s pain or distress or putting staff or other residents in harm’s way (57). Participants shared that doing what is best for the resident should drive decision-making, even if what is perceived as “best” includes elements of deception (eg, white lies) (58). However, resource constraints and organizational obstacles present significant barriers to the person-centered approach that is often required, and recommended as best practice (9,59,60). Other studies have reported similar experiences across licensed and unlicensed care staff, where organizational and systemic barriers deprioritize implementation of nonpharmaceutical interventions (23,28,61,62).

Lastly, participants situated their decision-making within perceived agency and authority. The human actors that participate in the situation of medication administration within AL/RC settings vary in proximity (ie, internal vs external) and power. We noticed a converse orientation between the perceived authority of an entity or individual and their proximity to medication administration with AL/RC settings. At the heart of medication administration within AL/RC are the resident receiving the medication and the staff person administering the medication. Prior studies have focused on the intersection of resident and staff autonomy when balancing safety, well-being, and choice regarding medication management in AL/RC (26,27,31,33). AL/RC residents have the right to refuse any treatment per resident rights recognized in regulatory practice (43), but the ability to assert this autonomy is influenced by cognitive capacity, care needs, staff perceptions of residents’ abilities, and AL/RC setting culture (6,31–33). Participants remarked on the difference in residents’ abilities to articulate needs and request medication based on whether they lived in a memory care environment. In some cases, there was evidence that AL/RC staff attempted to identify the cause of residents’ behavior and to start with nonpharmaceutical approaches. While we do not know from this study whether staff were trained to in the Antecedent–Behavior–Consequence model, prior work identified an association between ability to identify antecedents to behavior and less medication use (6). Future studies could take a systems approach (60) to examine staff practices, decision-making, training, and medication use in the context of policies, professional authority, and resident-centered care.

Long before a medication aide administers a PRN antipsychotic medication to a resident, other entities outside of the AL/RC setting context (eg, nonlocal) have made numerous decisions, and direct care staff must work within the parameters presented to them (59). We found that entities or individuals assigned with the most perceived authority (ie, Department of Human Services rule makers, physicians) were often external to daily resident care provision. Nurses and administrators oversee the writing of medication order parameters, ensuring direct care staff can administer medications and treatments without making assessment decisions. Prescribers write the original orders and generate access to the medications within AL/RC settings. Pharmacists or nurses review medication orders, recommending changes within the context of clinical decisions *and* regulatory compliance. Additionally, residents’ families present another human element with varying degrees of power over their loved one’s care, depending on their level of involvement and legal authority (eg, legal guardian, health proxy). Understanding the scope and context of APU within AL/RC settings necessitates a broader systems-level approach to this issue—beyond the medication pass.

### Limitations and Future Directions

This study has limitations worth considering for future research efforts. Long-term care settings, including AL/RC, have been disproportionately impacted by both resident morbidity and mortality and staffing shortage burdens as a direct result of COVID-19 (61,62). In addition to this trauma, pandemic-related restrictions limited recruitment and data collection efforts adversely affecting our ability to build the trust and relationships necessary with frontline care staff and administrators to gain buy-in for this study. Relying on remote recruitment through administrators and participants’ self-selection into this study during a global pandemic severely impacted participation. Research teams interested in conducting interviews or focus groups with the AL/RC workforce should consider investing in strategies that prioritize building relationships with administrators and staff and collaborate on data collection designs that simultaneously mitigate burden and offer an opportunity for participants to share their experiences.

The 11 study participants might not represent all AL/RC staff experiences and/or contexts. These participants delivered rich, deep interviews, but key experiences involving PRN APU might be missing based on who had the capacity to participate and our decision to interview only those who administer or oversee medication use in AL/RC communities. This study captures the views of diverse actors related to medications in AL/RC, including direct care staff, nurses, administrators, and consultant pharmacists. Future studies should include interviews and observations with multiple staff from the same AL/RC to aid contextualization of decision-making around PRN antipsychotic medication use as well as other actors involved in APU such as residents, their family members, and prescribers.

## Conclusions and Implications

Roles related to caregiving, ethical considerations, and perceived agency inform decision-making on whether to use antipsychotic medications. Participants described costs/benefits associated with both PRN APU and nonpharmaceutical interventions when responding to AL/RC residents’ behavioral expressions. Participants’ experiences emphasize the interactions across multiple levels of care. Balancing regulatory goals and norms with resident-centered practices underscores the need for a system-level perspective, extending beyond direct care staff passing antipsychotic medications to residents.

## Supplementary Material

igac052_suppl_Supplementary_Material_S1

igac052_suppl_Supplementary_Material_S2
