# Supplementary material for: Beyond the Medication Pass: Attitudes, Ethics, Agency, and Antipsychotic Medications in Assisted Living/Residential Care
Source: Innov Aging. 2022 Aug 17;6(6):igac052. doi: 10.1093/geroni/igac052 (PMC9701061; doi:10.1093/geroni/igac052)
Supplement: igac052_suppl_Supplementary_Material_S1 [file igac052_suppl_supplementary_material_s1.docx]

# Supplement 1. Interview guide

Introduction: Older adults living with Alzheimer’s disease and related dementias are more likely to express certain behaviors. When living in an assisted living or residential care environment, it is often up to caregiving staff to respond to resident’s behaviors. You may be aware that antipsychotic medication use and administration in AL/RC settings is a public policy priority in Oregon from a safety, oversight, and quality perspective.

There is a lot of talk about antipsychotic medications, but we would like to know more about your experiences working with older adults in assisted living.

Can you start by telling me a little bit about your job and what your responsibilities are?

1. Thinking about residents who have lived in this community who express behaviors, can you tell me about a time when one of those residents was helped through successful management by you and other staff?
2. How about a resident whose behavioral expressions were so severe that you or your staff were not able to respond, can you tell me that story?

FOLLOW UP QUESTION BANK

- How do you know if an intervention works or does not work when responding to residents’ agitation, anxiety, or distress?
- What types of training have you had on medication administration in older adults?
- Please describe a situation when you administered a PRN antipsychotic medication.
- How did you decide to administer this antipsychotic medication? Was there anything you would have done differently?
- What procedures or protocols does your facility/community/company have on record for the use of antipsychotic medications?
- If a resident has an order for an antipsychotic medication, what are you looking for when reviewing their Medication Administration Record (MAR)?
- How has the COVID-19 pandemic affected medication administration at this community?
- Let’s imagine the state proposes a rule that no more than 20% of residents can have an as needed antipsychotic medication. How would that affect your work
