# Supplementary material for: Beyond the Medication Pass: Attitudes, Ethics, Agency, and Antipsychotic Medications in Assisted Living/Residential Care
Source: Innov Aging. 2022 Aug 17;6(6):igac052. doi: 10.1093/geroni/igac052 (PMC9701061; doi:10.1093/geroni/igac052)
Supplement: igac052_suppl_Supplementary_Material_S2 [file igac052_suppl_supplementary_material_s2.docx]

Supplement 2. Ordered situational map of antipsychotic medication use in Oregon assisted living/residential care communities.

| **Individual Human Elements/Actors**  e.g., key individuals and significant (unorganized) people in the situation | **Nonhuman Elements/Actants**  e.g., technologies; material infrastructures; specialized information and/or knowledges; material “things” | | | | | | | | | | **Collective Human Elements/Actors**  e.g., particular groups; specific organizations |
| --- | --- | --- | --- | --- | --- | --- | --- | --- | --- | --- | --- |
| Medication technician | Challenging behavior | | | Staff: resident ratios | | | Creativity | | | | Family members |
| Psych provider | Scheduled medications | | | Clinical decision making | | | Teamwork | | | | Staff (generally) |
| Administrator | PRN medications | | | Chronic mental illness | | | Communication | | | | Health providers/clinicians |
| Resident(s) | Dosage | | | Teepa Snow's Positive Approach to Care | | | Bias/Stigma around medication use | | | | non-licensed staff |
| Primary care provider | Peace | | | Regulations | | | Comfort | | | | Board of Directors |
| Prescriber | Calm | | | Social model of care | | | Distress | | | | Relias |
| Consultant pharmacist | Nonpharmacologic intervention | | | Quality measures | | | Progress notes | | | | Consultant pharmacy services |
| Caregiver/universal worker | Urinary tract infection | | | Citations | | | Family involvement (or lack thereof) | | | | Oregon Health Authority |
| Surveyor | stability | | | Indications | | | Ethics | | | | Oregon Health Care Association |
| Nurse | person-centeredness | | | Dementia | | | Relationship building | | | | Oregon Partnership for Dementia Care |
| Licensed professional nurse | staff turnover | | | Neuropsychiatric symptoms | | | Managerial constraints | | | | CMS |
| Social worker | Medication administration policy | | | Gradual dose reduction | | | Antipsychotic | | | | Corporate long-term care |
| Mental health worker | PRN parameters | | | Medication administration record | | | Benzodiazepine | | | | FDA |
| Police | Care/service planning | | | Chemical restraint | | | Antidepressant | | | | American Medical Association |
| Medic/EMTs | Facility/community culture | | | FDA black box warning | | | Psychotropics | | | | Oregon Care Partners |
| Resident's Spouse/Child | Documentation/charting | | | Alcohol/substance use | | | Narcotics | | | | Crisis teams |
|  | Care transitions | | | Fear | | | Opioids | | | | Department of Human Services |
|  | Training/Education | | | Worry | | | Pain | | | |  |
| **Discursive Construction of Human Actors**  As found in the situation | | | | | | | | | | | |
| Need to know residents as people | | | | | | So used to resident behaviors we become complacent, charting does not always reflect experience | | | | | |
| Trying to make it person-centered | | | | | | Providers give multiple PRNs for the same issue, how are unlicensed staff supposed to distinguish | | | | | |
| Pharmacist role to tease out when medicine is really necessary, goal is to reduce | | | | | | Medication techs as team leaders/acting supervisors | | | | | |
| PCPs and prescribers generally lack geriatric knowledge | | | | | | Giving medications is a big responsibility | | | | | |
| In dementia, behaviors wax and wane, prescribers don't want to reduce these medications because of perceived stability | | | | | | Doctors are knowledgeable, know what they are doing and acting in best interests of residents | | | | | |
| A lot of staff don't have the background to be a med aide | | | | | | Caregivers as the "eyes and ears" / "detectives" | | | | | |
| Staff have a tough job to do | | | | | | AL residents can vocalize, tell you their needs, when needs are met, and request medications. Memory care is different | | | | | |
| Assumption that if resident did not have antipsychotic, they would be out of control | | | | | | Residents' feelings are valid, need to be listened to | | | | | |
| Who can self-direct and who cannot? | | | | | | Residents exhibit behaviors because they can't express emotions in other ways | | | | | |
| nurse responsible for medication administration training | | | | | | RN can't be a part of every decision | | | | | |
| "There are people who would rather give a pill than deal with residents" | | | | | | Caregivers as family | | | | | |
| Doctors order medication so the person fits the setting rather than ensuring residents are in the right setting for their needs | | | | | | Unlicensed caregivers as the "bottom rung" | | | | | |
| Need to know residents as people | | | | | | "Keep residents in a state of what we want them to be" | | | | | |
| Trying to make it person-centered | | | | | | Are people with ADRD allowed to display their disease? | | | | | |
| Pharmacist role to tease out when medicine is really necessary, goal is to reduce | | | | | | Residents respond to "vibes of caregivers and other residents" | | | | | |
| Some nurses go immediately to medication, others wait | | | | | |  | | | | | |
|  | | | | | |  | | | | | |
| **Discursive Construction of Nonhuman Actants**  As found in the situation | | | | | | | | | | | |
| Facility as home | | | | | | Medication record software in AL is not as robust | | | | | |
| Care transitions as triggering | | | | | | No medications to effectively treat NPS | | | | | |
| Overmedication vs. undermedication | | | | | | Facilities that do it right have people wandering, engaged, and not falling. | | | | | |
| Finding a balance | | | | | | Antipsychotics are used so commonly in memory care that you would think they are indicated to treat dementia and they are not | | | | | |
| Medications should be used correctly | | | | | | Meeting resident needs increases care quality | | | | | |
| Medication as last resort | | | | | | Clear medication orders prevent overmedicating | | | | | |
| Regulations have unintended consequences; skilled settings are seeing unintended consequences from regulations that were supposed to help | | | | | | Laws prevent staff from abusing residents | | | | | |
| Lot of work to determine why medications are started and determine root causes of behavior | | | | | | Can of worms | | | | | |
| Assisted living lacks regulations and data | | | | | | Some places abuse meds and residents by zonking them out | | | | | |
| Really clear parameters help non-nurses | | | | | | Assisted living is a gray area | | | | | |
| Staffing is a primary barrier to person-centered care because one-on-one interventions take time. | | | | | | Antipsychotics are indicated for psychiatric disorders such as schizophrenia, they don't do anything for dementia and operate as a chemical restraint | | | | | |
| Interdisciplinary team approach to CBC | | | | | |  | | | | | |
| Medication as weighted, emotional topic | | | | | |  | | | | | |
| Every situation is different | | | | | |  | | | | | |
|  | | | | | |  | | | | | |
|  | | | | | |  | | | | | |
| **Political/Economic Elements**  e.g., the state; particular industry/ies; local/regional/global orders; political parties; NGOs; politicized issues | | | | | | **Sociocultural/Symbolic Elements**  e.g., religion; race; sexuality; gender; ethnicity; nationality; logos; icons; other visual and/or aural symbols | | | | | |
| One on one interventions take time and money | | Desire for minimum state requirement for staffing | | | | Who gets included in care planning decisions? | | | | Positioning of the self- what would **I want** | |
| Generic Haldol or Ativan is pennies per tablet, much cheaper than hiring more staff, maximize returns | | Not allowed to restrain residents, meds seem like a restraint | | | | Clinician approach | | | | Whose comfort and distress are at the center of decision making? | |
| Facilities are penalized but facilities do not write prescriptions. | | Can't force someone to take something they don't want to | | | | Medical toolkit | | | | Avoid giving PRN to avoid stigma | |
| AstraZeneca fined $600 million for pushing seroquel for sleep | | Laws in places to protect elders can do a disservice when they are too restrictive | | | | Comparison to nursing facilities | | | | Exhaust all options before giving med | |
| Can't have one person caring for 25 people at $12/hour | | If there was a regulation tying % use of antipsychotics to quality, who is in charge of the extra scrutiny, paperwork, and heartache?  If there was a regulation tying % use of antipsychotics to quality, who is in charge of the extra scrutiny, paperwork, and heartache? | | | | Memory care should not be "nice and quiet" | | | | What does behavior mean? | |
| CMS guidelines do not apply to physicians | |  |  |  |  | Consistent, right care team | | | | Strong medications are not good for health | |
| Regulations/rules come from a safety point of view, rules can be hard to follow in practice | |  |  |  |  | Using more medications than normal is a sign that resident needs are not being met. If needs are met, behaviors go away | | | | the care conference: who is involved, who is not involved, identify goals of care, how to accomplish, and identify risks/benefits | |
| Implementing a % threshold would impact admissions/intake decisions; change the definition of ability to meet care needs. Go from resident-centered to regulation centered | |  | | | | Trust between care team-resident-health provider | | | | Meds as protection | |
|  |  |  | | | | Root causes, why are residents acting the way they do? | | | | Handle situation by treating residents as human beings | |
|  |  |  | | | | Bias/stigma related to psychotropic medication use | | | | Qualification of unlicensed caregivers to have an opinion on this issue | |
|  | |  | | | |  | | | | "Different and bad sometimes mean the same thing" | |
| **Implicated/Silent Elements**  As found in the situation | | | **Major Issues/Debates**  As found in the situation | | | | | | | | |
| Residents’ experience with med administration (only secondhand) | | | Care transitions to the emergency department | | | | | Sometimes nonpharm is not enough | | | |
| Pharmaceutical industry | | | No role for PRN antipsychotic medications, “low hanging fruit,” aim to get reduce or get rid of | | | | | Application in the field is difference that what is discussed behind a desk | | | |
| Prescriber decision making | | | PRN antipsychotic med use should be rare | | | | | Haldol use for hospice | | | |
|  | | | Staffing is biggest challenge to quality care | | | | | If needs are being met, medications are meaningless | | | |
|  | | | Avoid penalizing facilities and make sure people who truly need antipsychotic medications get them | | | | | Non-licensed staff taught to believe we overmedication with psychotropics out of convenience; but too many nonpharmaceutical interventions can result in poor outcomes | | | |
| **Spatial Elements**  e.g., spaces in the situation; geographical aspects; local, regional, national, and global spatial issues | | | Thresholds for use are not resident-centered and should be defined by appropriateness | | | | | Having PRN prescription does not mean regular administration | | | |
| Emergency room | | | "Some memory care are not good at memory care" | | | | | Standard nonpharm interventions or resident-specific interventions | | | |
| Memory care | | | No reason for PRN antipsychotics except for hospice care, an anti anxiety like a benzodiazepine would be more appropriate | | | | | MC residents use antipsychotic and antianxiety medications for well-being if there are no meds there isn't quality of life | | | |
| Switching placement to higher level of care | | | Antipsychotics prescribed for sleep or insomnia is careless | | | | | People living with mid-late dementia, focus should be on comfort | | | |
| secured units | | | Prescribers are prescribing these medications out of convenience | | | | | Giving antipsychotic medications changes people | | | |
| Importance of being in the right setting | | | Dichotomies: right vs. wrong, bad vs. good, nonpharm vs. pharm, drooling in a chair vs. agitated | | | | | Antipsychotics are good for people with mental health issues but not for people with dementia | | | |
|  | | | Mandate physician training and oversight similar to opioids | | | | | Application in the field is difference that what is discussed behind a desk | | | |
|  | | | Non-licensed staff taught to believe we overmedication with psychotropics out of convenience; but too many nonpharmaceutical interventions can result in poor outcomes | | | | | Potential unintended consequence > overmedicating by transitioning to routine use of medications rather than trusting PRN functionality and staff | | | |
| **Temporal Elements**  e.g., historical, seasonal, crisis, and/or trajectory aspects | | | | | **Related Discourses**  e.g., normative expectations of actors, actants, and/or other specified elements; moral/ethical elements; mass media and other popular cultural discourses; situation-specific discourses | | | | | | |
| Resident exhibits behavior > attempt to calm >call wife>send to ER>UTI>treat and transition back to community | | | | | Non-licensed medication aides cannot assess | | | | "This job is more than a job. It requires compassion, patience, and sacrifice." | | |
| PRN used>how many times was it used?>what was the med?> what nonpharm interventions were attempted?>determine whether staff default to medication and why | | | | | Older people do not tolerate side effects of medications well | | | | AL interventions are cognitive, mental health, talk interaction while MC interventions are activity based | | |
| PRN not used> no use for 60+ days> discontinue | | | | | What is memory care? | | | | "We get residents other people will pass on" | | |
| OBRA 1987>heightened focus on antipsychotic medications>no significant changes since then | | | | | When are antipsychotic medications warranted? | | | | "Trainings pride themselves on not medicating" | | |
| New employee hired>get to know residents>take on medication responsibilities>mentored training and observation | | | | | Multiple antipsychotic medications used at once | | | | What would I want if I am in this situation? | | |
| Staff spend 15-16 hours a day with residents, lots of interaction | | | | | Medication as a stopgap | | | | Physicians clarify and are specific with parameters | | |
|  |  |  |  |  | Medications keep people in the community | | | | Recall of PRN medications often don't include actual antipsychotics (rather lorazepam) | | |
| If there is no relief after X amount of time, give medication | | | | | Non-licensed medication aides cannot assess | | | | Government needs to support staff more | | |
| Residents' health improves, medication orders change | | | | | Older people do not tolerate side effects of medications well | | | | Oregon no longer has psych hospital for older adults, lost valuable resource | | |
| Care plans are dynamic | | | | | What is memory care? | | | | Need for broader education on the aging process and dementia | | |
| Behavioral issues>staff attempts to interact with nonpharm>LPN brought in if interventions don't help>never offer antipsychotics in this situation | | | | | When are antipsychotic medications warranted? | | | | "We get residents other people will pass on" | | |
|  |  |  |  |  | Multiple antipsychotic medications used at once | | | | Meeting needs increases care quality | | |
|  | | | | | Medication as a stopgap | | | | Operate on group consensus | | |
|  | | | | | Medications keep people in the community | | | | PRN medication is a last resort | | |
